# Ki-67 (30-9) scoring and differentiation of Luminal A- and Luminal B-like breast cancer subtypes

**DOI:** 10.1007/s10549-019-05402-w

**Published:** 2019-08-17

**Authors:** Giuseppe Viale, Amy E. Hanlon Newell, Espen Walker, Greg Harlow, Isaac Bai, Leila Russo, Patrizia Dell’Orto, Patrick Maisonneuve

**Affiliations:** 1 Department of Pathology, IEO European Institute of Oncology IRCCS, Milan, Italy; 2 University of Milan, Milan, Italy; 3 Ventana Medical Systems, Inc., Tucson, AZ USA; 4 Division of Epidemiology and Biostatistics, IEO European Institute of Oncology IRCCS, Milan, Italy

**Keywords:** Ki-67, Luminal A, Luminal B, Differentiation, Biomarker

## Abstract

**Introduction:**

Ki-67 labeling index assessed by immunohistochemical assays has been shown useful in assessing the risk of recurrence for estrogen receptor (ER)-positive HER2-negative breast cancers (BC) and distinguishing Luminal A-like from Luminal B-like tumors. We aimed to assess the performance of the Ventana CONFIRM anti-Ki-67 (30-9) Rabbit Monoclonal Primary Antibody.

**Methods:**

We constructed a case–cohort design based on a random sample (*n* = 679) of all patients operated on for a first primary, non-metastatic, ER-positive, HER2-negative BC at the European Institute of Oncology (IEO) Milan, Italy during 1998–2002 and all additional patients (*n* = 303) operated during the same period, who developed an event (metastasis in distant organs or death due to BC as primary event) and were not included in the previous subset. Multivariable Cox proportional hazards regression with inverse subcohort sampling probability weighting was used to evaluate the risk of event according to Ki-67 (30-9) and derived intrinsic molecular subtype, using previously defined cutoff values, i.e., respectively 14% and 20%.

**Results:**

Ki-67 was < 14% in 318 patients (32.4%), comprised between 14 and 19% in 245 patients (24.9%) and ≥ 20 in 419 patients (42.7%). At multivariable analysis, the risk of developing distant disease was 1.88 (95% CI 1.20–2.93; *P* = 0.006) for those with Ki-67 comprised between 14 and 19%, and 3.06 (95% CI 1.93–4.84; *P* < 0.0001) for those with Ki-67 ≥ 20% compared to those with Ki-67 < 14%. Patients with Luminal B-like BC had an approximate twofold risk of developing distant disease (HR = 1.91; 95% CI 1.35–2.71; *P* = 0.0003) than patients with Luminal A-like BC defined using Ki-67 (30-9).

**Conclusions:**

Ki-67 evaluation using the 30-9 rabbit monoclonal primary antibody was able to stratify patients with ER-positive HER2-negative BC into prognostically distinct groups. Ki-67 assessment, with strict adherence to the international recommendations, should be included among the clinically useful biological parameters for the best treatment of patients with BC.

**Electronic supplementary material:**

The online version of this article (10.1007/s10549-019-05402-w) contains supplementary material, which is available to authorized users.

## Introduction

Ki-67 is a nuclear antigen expressed by all proliferating cells during late G1 through the M phases of the cell cycle, peaking in the G2-M and with a rapid decline after mitosis [1]. Ki-67 labeling index assessed by immunohistochemical assays is a powerful prognostic marker in breast cancer. It is especially useful in assessing the risk of recurrence for estrogen receptor (ER)-positive HER2-negative breast cancers, where it may be considered a surrogate of the molecular assays for distinguishing Luminal A-like from Luminal B-like tumors [2]. Despite methodological problems still exist in the determination of Ki-67 in the routine clinical practice, both the Panellists of the St. Gallen Consensus [3] and the European Group on Tumor Markers (EGTM) [4] have endorsed use of Ki-67 in combination with established prognostic factors for determining prognosis, especially if values are low (e.g. < 10% of immunostained tumor cells) or high (e.g. > 25% cell staining). The higher cutoff value is based on a meta-analysis showing that a threshold of > 25% cell staining was associated with a greater risk of death compared with lower values [5].

The mouse monoclonal antibody MIB-1 was the first antibody to be raised against a formalin-resistant epitope of Ki-67, and it has been extensively used in both clinical research and routine practice [6].

We evaluated the performance of the CONFIRM anti-Ki-67 (30-9) Rabbit Monoclonal Primary Antibody in assessing the risk of distant relapses in a large series of patients with ER-positive HER2-negative breast cancer treated and followed up in a single Institution.

## Materials and methods

### Patients selection

The initial cohort (9415 patients) comprised all women operated on for a first primary, non-metastatic, ER-positive HER2 negative, breast cancer (BC) at the European Institute of Oncology (IEO) Milan, Italy, who did not receive neoadjuvant treatment [7]. We subsequently restricted the cohort to 3986 patients operated on between 1998 and 2002 and for whom long-term follow-up data were available. A case–cohort [8] was built by randomly selecting approximately 17% of the above cohort (*n* = 679). Additional patients (*n* = 303) who developed an event (metastasis in distant organs or death due to BC as primary events) were added to this cohort (Supplementary Fig. 1).

### Laboratory methods

Ki-67 was evaluated using the VENTANA CONFIRM anti-Ki-67 (30-9) Rabbit Monoclonal Primary Antibody (Ventana Medical Systems, Inc., Tucson, AZ) using OptiView IHC DAB detection on the BenchMark ULTRA advanced staining platform. The stained slides were evaluated at the IEO by certified pathologists trained to score Ki-67 according to the recommendations of the International Ki-67 in Breast Cancer Working Group [9].

Samples were retrieved from the Pathology archives with Institutional Review Board approval and classified as ‘Luminal A-like’; estrogen receptor (ER)-positive, HER2-negative tumors with “low” Ki-67 (< 14%) or with “intermediate” Ki-67 (14–19%) and “high” progesterone receptor expression (PgR ≥ 20%), and ‘Luminal B-like’; HER2 negative, tumors ER positive, HER2 negative, with “intermediate” Ki-67 (14–19%) and “low” PgR (< 20%) or with “high” Ki-67 (≥ 20%) [7] (Supplementary Table 1).

### Statistical methods

Associations between clinicopathological characteristics and Ki-67 expression were evaluated with the Mantel–Haenszel test for trend. The main outcome was distant disease-free survival (DDFS) and was calculated from the date of surgery to the date of any first event or the date of last contact with the patient. Distant disease was defined as the occurrence of metastasis in distant organs or death due to BC as primary events. The rate of events in the subcohort was calculated dividing the number of events recorded during follow-up by the total number of patient-years accumulated during the observation period and 95% confidence intervals calculated using the mid-P exact method. Cumulative incidence curves were drawn for patients in the subcohort using the Kaplan–Meier method and difference between patient subgroups was assessed using the log-rank test. Multivariable Cox proportional hazards regression with inverse subcohort sampling probability weighting was used to evaluate the risk of metastasis or death from BC across groups in the combined case–cohort [10, 11]. In the multivariable analysis, Ki-67 was considered either as a continuous variable, expressing the hazard ratio (HR) for each 10% increase of Ki-67 labeling index or was categorized using the same cutoff values used for the definition of surrogate BC molecular subtypes, i.e., respectively 14% and 20% [7]. Other variables considered in the multivariable analysis include menopausal status, pathological T (pT1, pT2, pT3/4), regional lymph node status (pN0, pN+), tumor grade (G1, G2, G3), peritumoral vascular invasion (PVI) (absent, present), estrogen receptor (ER) and progesterone receptor (PgR) expression (< 20% vs. ≥ 20%) and adjuvant treatment (none, hormone therapy alone, or hormone therapy plus chemotherapy). Finally, restricted cubic spline Cox regression models were applied to assess dose–response relationships. Analyses were carried out with the SAS software (version 9.4, Cary NC). *P* values were two sided. *P* < 0.05 were considered statistically significant.

## Results

The case–cohort comprised 982 patients (679 patients are part of the 1998–2002 subcohort (including 84 with event) and 303 are patients who developed event outside of the subcohort (Supplementary Fig. 1). Of the 387 events, 10 were death from breast cancer as a first event, and the remaining 377 were distant metastases, with a prevalence of bone metastases (155 events) followed by lung (46 events) and liver metastases (44 events). Ninety-five patients developed multiple metastases as first event.

Distribution of Ki-67 according to clinicopathological characteristics is displayed in Table 1. Overall, the median Ki-67 expression was 18%, in 318 patients (32.4%) it was < 14%, in 245 patients (24.9%) it was comprised between 14 and 19% and in 419 patients (42.7%) it was ≥ 20%. Distribution was significantly different across all subgroups evaluated, Ki-67 expression being significantly higher in premenopausal women *P* = 0.0002), and being directly associated with pT, pN, tumor grade, presence of PVI, and inversely associated with the expression of ER and PgR receptors (*P* < 0.0001 for all the associations). Particularly elevated median Ki-67 expression (27%) was found in patients with poorly differentiated tumors and in patients with tumors showing low (1–19% immunoreactive tumor cells) or moderate (20–49% immunoreactive tumor cells) ER expression, and with a median Ki-67 value of 35% and 27%, respectively. Lowest median Ki-67 was observed in patients with well-differentiated tumors (median 9%).

**Table 1:** Distribution of Ki-67 (30-9) according to selected clinicopathological characteristics

	Patients	Ki-67 (30-9)					
		Median (25th–75th)	Mean Std. Dev	< 14% N (%)	14–19% N (%)	≥ 20% N (%)	*P* value*
All	982	18 (12–25)	19.6 ± 11.9	318 (100)	245 (100)	419 (100)	
Menopausal status							
Pre/peri	434	19 (13–26)	21.2 ± 12.6	115 (36.2)	109 (44.5)	210 (50.1)	
Post	548	16 (11–24)	18.4 ± 11.1	203 (63.8)	136 (55.5)	209 (49.9)	0.0002
pT							
pT1	590	15 (10–22)	16.9 ± 10.2	245 (77.0)	160 (65.3)	185 (44.2)	
pT2	343	22 (16–29)	24.3 ± 13.4	61 (19.2)	72 (29.4)	210 (50.1)	
pT3/4	49	19 (14–25)	19.7 ± 8.5	12 (3.8)	13 (5.3)	24 (5.7)	< 0.0001
pN							
pN0	459	16 (10–22)	17.7 ± 12.5	195 (61.3)	107 (43.7)	157 (37.5)	
1–3 Positive nodes	292	18 (13–25)	20.0 ± 11.2	81 (25.5)	86 (35.1)	125 (29.8)	
≥ 4 Positive nodes	212	22 (16–29)	23.3 ± 10.2	35 (11.0)	48 (19.6)	129 (30.8)	< 0.0001
pNx	19	16 (7–25)	19.5 ± 14.5	7 (2.2)	4 (1.6)	8 (1.9)	
Grade							
G1	181	9 (5–14)	10.5 ± 6.6	131 (41.2)	34 (13.9)	16 (3.8)	
G2	489	16 (12–21)	17.0 ± 8.0	169 (53.1)	170 (69.4)	150 (35.8)	
G3	286	27 (22–34)	30.1 ± 13.0	8 (2.5)	35 (14.3)	243 (58.0)	< 0.0001
Unknown	26	17 (10–24)	17.3 ± 7.8	10 (3.1)	6 (2.4)	10 (2.4)	
PVI							
Absent	672	16 (10–23)	17.8 ± 11.3	263 (82.7)	169 (69.0)	240 (57.3)	
Present	310	22 (16–28)	23.5 ± 12.2	55 (17.3)	76 (31.0)	179 (42.7)	< 0.0001
ER							
1–19%	17	35 (28–50)	38.7 ± 19.5	0 (0.0)	4 (1.6)	13 (3.1)	
20–49%	61	27 (17–30)	25.2 ± 13.0	13 (4.1)	12 (4.9)	36 (8.6)	
≥ 50%	904	19 (12–27)	21.6 ± 13.5	305 (95.9)	229 (93.5)	370 (88.3)	< 0.0001
PgR							
1–20%	309	21 (14–30)	23.2 ± 14.2	86 (27.0)	75 (30.6)	148 (35.3)	
20–49%	180	22 (14–29)	22.9 ± 13.3	52 (16.4)	38 (15.5)	90 (21.5)	
≥ 50%	493	18 (12–26)	21.2 ± 13.6	180 (56.6)	132 (53.9)	181 (43.2)	0.001
Adjuvant treatment							
None	41	12 (6–19)	15.0 ± 13.3	25 (7.9)	6 (2.4)	10 (2.4)	
Hormone therapy	470	14 (10–21)	15.8 ± 9.4	217 (68.2)	119 (48.6)	134 (32.1)	
Chemotherapy	470	22 (16–29)	23.9 ± 12.5	76 (23.9)	120 (49.0)	274 (65.4)	< 0.0001

*P-value based on the Mantel–Haenszel Chi square test for trend

**Fig. 1:**
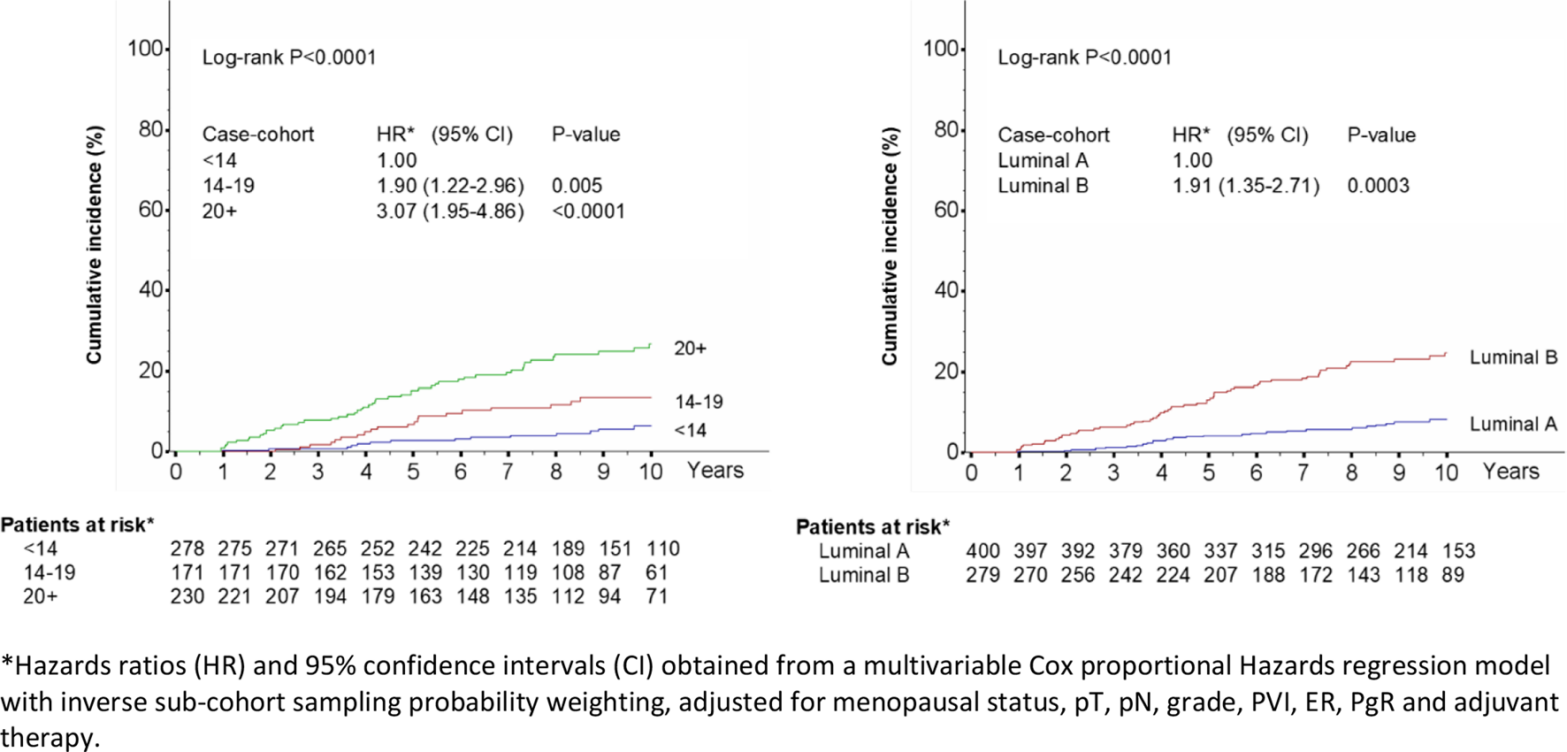
Cumulative incidence of events in the subcohort (*N* = 679) and corresponding Hazards Ratios in the case–cohort (*N* = 982) according to Ki-67 (30-9) and derived intrinsic molecular subtype

In the subcohort, 84 patients developed distant disease or died from BC as first event during follow-up, corresponding to an event rate of 1.60 per 100 patient-year (Table 2). The event rate increased from 0.61 per 100 patient-year for patients with low Ki-67 (< 14%), to 1.47 per 100 patient-year for those with intermediate Ki-67 (14–19%) and to 3.12 per 100 patient-year for those with Ki-67 ≥ 20%. The 10-year cumulative incidence of distant metastasis (or BC-related death as first event) according to categories of Ki-67 is shown in Fig. 1. The event rate was about constant over time in the three groups. At 5 years and 10 years, respectively, 2.7% (95% CI 1.3–5.5) and 6.4% (95% CI 3.8–10.7) of patients with Ki-67 < 14% developed an event against 6.8% (95% CI 3.8–11.9) and 13.5% (95% CI 8.9–20.3) of those with intermediate Ki-67, and 15.2% (95% CI 11.0–20.8) and 26.6% (95% CI 20.7–33.8) of those with high Ki-67.

**Table 2:** Distribution of events and multivariable analysis

	Sub–cohort (*n* = 679)			Additional cases (*n* = 303)	Case–cohort (*n* = 982)	
	Patients	Events	Event rate per 100 patient-year (95% CI)		HR (95% CI)^a^	*P*-value
Ki-67 (30-9)^b^						
< 14%	278	14	0.61 (0.35–1.00)	40	1.00	
14–19%	171	20	1.47 (0.92–2.23)	74	1.88 (1.20–2.93)	0.006
≥ 20%	230	50	3.12 (2.34–4.08)	189	3.06 (1.93–4.84)	< 0.0001
Menopausal status						
Pre/peri	289	34	1.50 (1.05–2.07)	145	1.00	
Post	390	50	1.68 (1.26–2.20)	158	1.43 (0.82–2.49)	0.20
pT						
pT1	466	34	0.88 (0.62–1.22)	124	1.00	
pT2	191	44	3.51 (2.58–4.67)	152	1.75 (1.24–2.45)	0.001
pT3/4	22	6	4.41 (1.79–9.18)	27	3.26 (1.66–6.39)	0.0006
pN						
pN0	378	18	0.57 (0.35–0.89)	81	1.00	
pN+	402	64	3.22 (2.50–4.09)	121	1.86 (1.25–2.78)	0.002
Grade						
G1	166	6	0.42 (0.17–0.87)	15	1.00	
G2	343	36	1.36 (0.96–1.86)	146	1.90 (1.06–3.39)	0.03
G3	151	40	3.91 (2.83–5.28)	135	2.43 (1.27–4.65)	0.008
PVI						
Absent	510	45	1.10 (0.81–1.46)	162	1.00	
Present	169	39	3.36 (2.42–4.55)	141	1.53 (1.11–2.12)	0.009
ER						
≥ 20%	665	81	1.57 (1.26–1.94)	300	1.00	
< 20%	14	3	3.06 (0.78–8.33)	3	1.05 (0.43–2.61)	0.91
PgR						
≥ 20%	474	53	1.45 (1.10–1.88)	199	1.00	
< 20%	205	31	1.95 (1.35–2.73)	104	1.01 (0.72–1.41)	0.98
Adjuvant treatment						
None	34	3	1.22 (0.31–3.33)	7	1.50 (0.61–3.69)	0.37
Hormone therapy	382	20	0.64 (0.39–0.94)	88	1.00	
Chemotherapy	262	61	3.18 (2.45–4.06)	208	1.73 (1.11–2.69)	0.02

^a^Hazards ratio (HR and 95% confidence intervals (CI) obtained from Cox proportional Hazards regression with inverse subcohort sampling probability weighting as defined in Miettinen (1976) using the SAS macro CCREGRESSION provided by Kulathinal et al. (2007)

^b^Results of an alternative multivariable model with Ki-67 (30-9) set as a continuous variable shows HR = 1.25 (95% CI 1.08–1.44; *P* = 0.002) for a 10% increase Ki-67, adjusted for menopausal status, pT, pN, grade, PVI, ER, PgR and adjuvant therapy

**Fig. 2:**
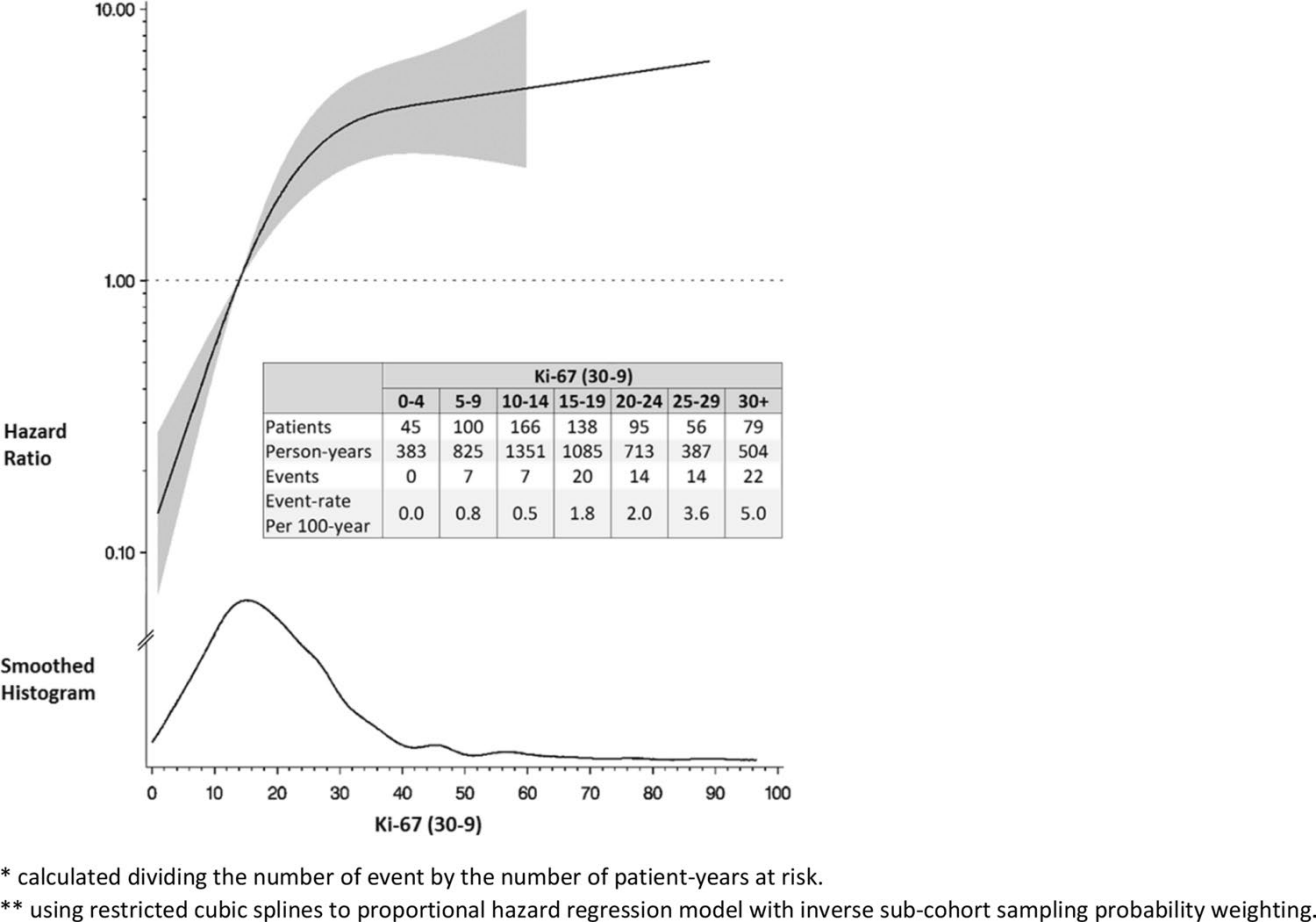
Event rate* in the subcohort and Hazard Ratio** for distant disease-free survival according to Ki-67 (HR set to 1.00 for Ki-67 = 14%) in the case–cohort

Dose–response in the subcohort was further evaluated in a plot based on a restricted cubic spline Cox regression model (Fig. 2): the rate of events increased linearly with the increasing expression of Ki-67 for values comprised between 0 and 30%. Above this threshold, the rate of events increased only slightly, and was based on a small fraction of the patients (only 79 (12%) patients in the subcohort had Ki-67 ≥ 30%).

In the case–cohort, including additional cases reported outside of the subcohort and after adjusting for other prognostic factors (menopausal status, pT, pN, grade, PVI, ER, and PgR) and for the type of adjuvant treatment received, Ki-67 remained significantly associated with DDFS. The relative risk of developing distant disease was 1.88 (95% CI 1.20–2.93; *P* = 0.006) for those with Ki-67 comprised between 14 and 19%, and 3.06 (95% CI 1.93–4.84; *P* < 0.0001) for those with Ki-67 ≥ 20% compared to those with Ki-67 < 14%. Other independent prognostic factors include pT, pN, tumor grade, PVI. Patients receiving chemotherapy were also at higher risk of events (Table 2).

We used previously published criteria for the definition of surrogate BC molecular subtype using Ki-67 (Supplementary Table 1). In the subcohort, 400 (58.9%) patients were classified as having “luminal A-like” and 279 (41.1%) “luminal B-like” BC. The 5-year and 10-year cumulative incidence of distant metastasis (or BC-related death as first event) were respectively 4.2% (95% CI 2.6–6.8) and 8.2% (95% CI 5.7–11.9) in the Luminal A group and 13.2% (95% CI 9.6–17.9) and 24.5% (95% CI 19.4–30.8%) in the Luminal B group (log-rank *P* < 0.0001) (Fig. 1).

In the whole case–cohort, multivariable analysis confirmed statistically significant increased risk of events for women with “Luminal B-Like” BC compared to women with “Luminal A-Like“ BC (HR = 1.91; 95% CI 1.35–2.71; *P* = 0.0003), after adjustment for menopausal status, pT, pN, grade, PVI and adjuvant therapy (Table 3).

**Table 3:** Distribution of events and multivariable analysis

	Sub–cohort (*n* = 679)			Additional cases (*n* = 303)	Case–cohort (*n* = 982)	
	Patients	Events	Event rate per 100 patient-year (95% CI)		HR (95% CI)^a^	*P*-value
Molecular subtype						
Luminal A-like	400	27	0.83 (0.56–1.20)	88	1.00	
Luminal B-like	279	57	2.84 (2.17–3.65)	215	1.91 (1.35–2.71)	0.0003

^a^Hazards ratio (HR and 95% confidence intervals (CI) obtained from multivariable Cox proportional Hazards regression with inverse subcohort sampling probability weighting as defined in Miettinen (1976) using the SAS macro CCREGRESSION provided by Kulathinal et al. (2007), adjusted for menopausal status, pT, pN, grade, PVI and adjuvant therapy

## Discussion

Ki-67 labeling index is a clinically validated prognostic factor in early breast cancer. In the neoadjuvant setting, it predicts the likelihood of pathological complete response (pCR) to chemotherapy. Furthermore, Ki-67 in the residual tumor [12, 13], and changes of Ki-67 labeling index between primary and residual tumors are prognostic for long-term outcome [14, 15].

Decline of Ki-67 after few weeks of neoadjuvant endocrine therapy is correlated with a better long-term outcome of ER-positive HER2-negative disease [16] and Ki67 assessment in the residual tumor after neoadjuvant endocrine treatment is predictive of long-term outcome [17]. In the adjuvant setting, Ki-67 is a prognostic marker for disease-free and overall survival independent of tumor stage [18, 19].

Despite its undisputed prognostic value, however, Ki-67 labeling index per se is not predictive of the benefit of adding chemotherapy to endocrine therapy in the treatment of patients with ER-positive HER2-negative early breast cancer [20]. To inform the choice of systemic treatment for these patients, Ki-67 labeling index should be used in combination with other parameters, including tumor grade and a quantitative evaluation of ER and progesterone receptor (PgR) expression [3]. This has also been endorsed by the updated guidelines from the European Group of Tumor Markers [4].

By using a similar multifactorial approach, we have previously proposed a surrogate immunohistochemical definition of Luminal A-like and Luminal B-like breast cancer [7] that could be helpful in tailoring the systemic treatment, especially when multiparameter molecular assays are not available.

Most of the aforementioned studies have been conducted using the MIB-1 monoclonal antibody to Ki-67. Here, we have shown that Ki-67 evaluation using the Ventana 30-9 rabbit monoclonal primary antibody, was similarly able to stratify patients with ER-positive HER2-negative breast cancer into prognostically distinct groups. Ki-67 evaluation in this cohort enabled maximizing the number of patients classified as having ‘Luminal A-like’ intrinsic subtype for whom the use of cytotoxic drugs could be at large avoided. Indeed, 400 (58.9%) patients in the subcohort were classified as having “Luminal A-like” and 279 (41.1%) “Luminal B-like” BC. These figures are strikingly similar to those obtained by Cheang and colleagues [21] using a rabbit monoclonal antibody to Ki-67 (clone SP6) in a series of 2847 hormone receptor-positive breast carcinomas, and showing a 59% prevalence of Luminal A-like tumors. More recently, a study evaluating Ki-67 with the MIB-1 antibody in a series of 4718 patients with hormone receptor-positive disease also found a prevalence of Luminal A-like tumors of 58.2% [22]. Currently, the scientific community is still concerned about a perceived lack of accuracy and reproducibility in the assessment of Ki-67 in the clinical setting. Major steps toward a harmonization of Ki-67 scoring in breast cancer, however, have been already made [9, 23–25], and it may be predicted that Ki-67 assessment, with strict adherence to the international recommendations, will ultimately be included among the clinically useful biological parameters for the best treatment of patients with breast carcinoma.

## Electronic supplementary material

Below is the link to the electronic supplementary material.

Supplementary material 1 (DOCX 47 kb)
