# Supplementary material for: Ki-67 (30-9) scoring and differentiation of Luminal A- and Luminal B-like breast cancer subtypes
Source: Breast Cancer Res Treat. 2019 Aug 17;178(2):451–8. doi: 10.1007/s10549-019-05402-w (PMC6797656; doi:10.1007/s10549-019-05402-w)
Supplement: Supplementary file 1 — Supplementary material 1 (DOCX 47 kb) [file 10549_2019_5402_MOESM1_ESM.docx]

**Supplementary Table 1**. Definition of Intrinsic molecular subtype

| **Intrinsic subtype** | **Clinico-pathologic surrogate definition** |
| --- | --- |
| Luminal A | **‘Luminal A-like’**  *all of:* ER positive  HER2-negative *and at least one of:*  Ki-67 “low” (<14%)  Ki-67 “intermediate” (14-19%) and PgR “high” (≥20%) |
| Luminal B  (HER2-Negative) | **‘Luminal B-like (HER2 negative)’**  *all of:* ER positive  HER2-negative  *and at least one of:*  Ki-67 “intermediate” (14-19%) and PgR “negative or low” (<20%)  Ki-67 “high” (≥20%) |

Abbreviations: ER, estrogen receptor; PgR, progesterone receptor

**Supplementary Figure 1**. Patients’ selection

**
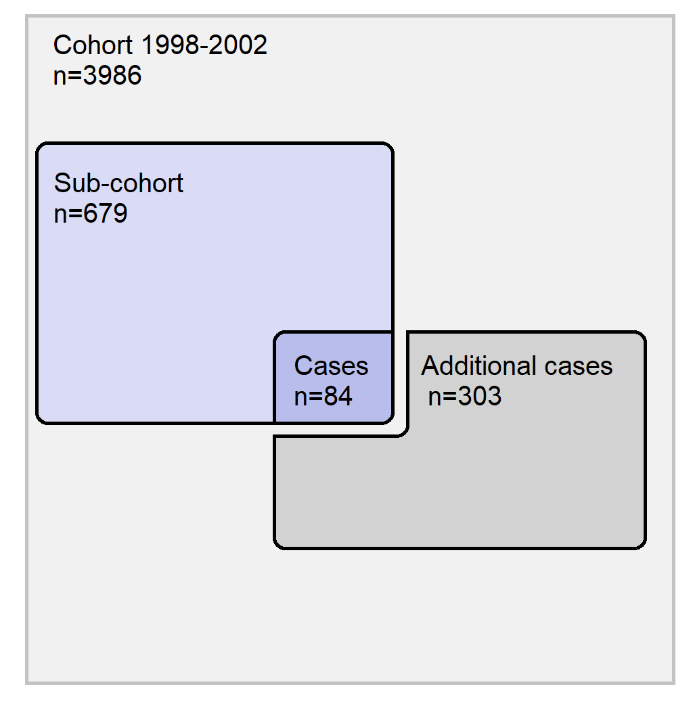
**
